# The pathophysiological nature of sarcomeres in trigger points in patients with myofascial pain syndrome: A preliminary study

**DOI:** 10.1002/ejp.1647

**Published:** 2020-09-10

**Authors:** Feihong Jin, Yaqiu Guo, Zi Wang, Ahmed Badughaish, Xin Pan, Li Zhang, Feng Qi

**Affiliations:** ^1^ Department of Anesthesiology and Pain Clinic Qilu Hospital, Cheeloo College of Medicine, Shandong University Ji’nan China; ^2^ Department of Anesthesiology Jinan Maternity and Child Care Hospital Ji’nan China; ^3^ Department of Anesthesiology First Affiliated Hospital of Shandong TCM University Ji’nan China; ^4^ Department of Orthopedics Qilu Hospital, Cheeloo College of Medicine, Shandong University Ji’nan China

## Abstract

**Background:**

Myofascial pain syndrome (MPS) has a high global prevalence and is associated with myofascial trigger points (MTrPs) in taut bands or nodules. Little is known about the aetiology. The current study assessed the pathophysiological characteristics of MTrPs in MPS patients.

**Methods:**

Biopsies of the trapezius muscle were collected from the MTrPs of MPS patients (MTrP group; *n* = 29) and from healthy controls (control group; *n* = 24), and their morphologies were analysed via haematoxylin‐eosin (H&E) and Masson staining. A protein microarray was used to detect the receptor tyrosine kinase (RTK) family proteins. mRNA and long non‐coding RNA (lncRNA) sequencing and analysis were conducted, and immunohistochemistry and Western blotting were used to examine the expression of EphB and Rho family proteins.

**Results:**

Abnormally contracted sarcomeres showed enlarged, round fibres without inflammation or fibrosis. An lncRNA‐mRNA network analysis revealed activation of muscle contraction signalling pathways in MTrP regions. Among RTK family proteins, 15 exhibited increased phosphorylation, and two exhibited decreased phosphorylation in the MTrP regions relative to control levels. In particular, EphB1/EphB2 phosphorylation was increased on the muscle cell membranes of abnormal sarcomeres. RhoA and Rac1, but not cell division control protein 42 (Cdc42), were activated in the abnormal sarcomeres.

**Conclusions:**

EphB1/EphB2 and RhoA/Rac1 might play roles in the aetiology of abnormally contracted sarcomeres in MTrPs without inflammatory cell infiltration and fibrotic adhesion.

**Significance:**

Contracted sarcomeres were found in MTrP regions, which is consistent with the MTrP formation hypothesis. EphB1/EphB2 and RhoA/Rac1 might play roles in the sarcomere contractile sites of MTrPs, which may be promising therapeutic targets.

## 1 INTRODUCTION

Musculoskeletal pain is a major cause of human suffering worldwide, with a prevalence between 13.7% and 47% among the global population (Bergman et al., 2001; Cimmino, Ferrone, & Cutolo, 2011). Myofascial pain syndrome (MPS) is one of the most common types of musculoskeletal pain. The presence of myofascial trigger points (MTrPs) is necessary for diagnosing MPS and is not only important in the pathogenesis of MPS but also significant in clinical treatment sites.

An MTrP is defined as a hyperirritable tender spot in a taut band (TB) that can be palpated in skeletal muscle (Alvarez & Rockwell, 2002; Borg‐Stein & Simons, 2002). Several previous studies have reported morphological changes in patients with MPS, such as jagged red fibres (Larsson, Bengtsson, Bodegård, Henriksson, & Larsson, 1988), moth‐eaten fibres (Windisch et al., 1999) or giant circular muscle fibres (Simons & Travell, 1999). An increasing amount of evidence supports the notion that MTrPs in TBs are associated with abnormally contracted sarcomeres within muscle fibres. In a previous study (Simons & Stolov, 1976), researchers obtained a microphotograph of a ‘contraction knot’ within a TB in the muscle of a canine, and Akamatsu and colleagues biopsied MTrPs from fresh cadavers and observed wider A‐bands, as well as I‐band loss (Akamatsu et al., 2015). In addition, some studies have attempted to reproduce an MTrP region in muscle. Histopathological changes in MTrPs in a rat model (Zhang et al., 2017) and intramuscular injection of acetylcholinesterase (Mense, Simons, Hoheisel, & Quenzer, 2003) in skeletal muscle of rat have revealed abnormally contracted sarcomeres, which have never been confirmed or replicated in humans. Thus, the existence of and the mechanism through which abnormally contracted sarcomeres are formed remains controversial.

Many hypotheses regarding the pathogenesis of MTrPs have been proposed. Testing of an integrated hypothesis in a previous study (Gerwin, Dommerholt, & Shah, 2004) demonstrated that abnormally contracted sarcomeres are caused by aberrant depolarization of the motor end‐plate mediated by acetylcholine (Ach), and persistent muscle contraction can lead to a local ATP crisis, which may evoke the release of neuroreactive substances and metabolic byproducts (i.e. bradykinin, substance P and serotonin [5‐hydroxytryptamine]) that could sensitize afferent nerves and cause myalgia. However, the sustained release of Ach may not be necessary to maintain a trigger point. In a study (Ga, Koh, Choi, & Kim, 2007) comparing the efficacy of motor nerve blockade by intramuscular lidocaine injection and dry needle stimulation, the group receiving intramuscular stimulation with a dry needle exhibited a >40% improvement compared to the lidocaine injection group. Lidocaine decreases Ach release in a dose‐dependent manner (Post, Sarracino, Gergis, & Sokoll, 1982). Furthermore, the effect of botulinum toxin A on mechanical pain thresholds or pain intensity when injected into MTrP areas remains controversial (Ernberg, Hedenberg‐Magnusson, List, & Svensson, 2011). Another mechanism may involve positive feedback loops that maintain MTrPs. To gain an in‐depth understanding of the pathophysiological mechanisms of MTrP formation, it is helpful to consider the normal physiology of skeletal muscles.

In the intact body, the contraction of muscle fibres is primarily regulated by mechanical activation of receptors and the contractile proteins myosin and actin (Webb, 2003). Receptor tyrosine kinases (RTKs) constitute a large family of transmembrane receptors. Many studies have demonstrated that RTKs participate in the Ca^2+^‐sensitizing muscle contraction process (Norton, Broughton, Jernigan, Walker, & Resta, 2013). Ca^2+^ is known to be essential for muscle contraction, but the increase in the intracellular Ca^2+^ concentration is transient. The contractile response can be maintained by the Ca^2+^ sensitization mechanism mediated by Rho kinases – a group of small GTP‐binding proteins (Puetz, Lubomirov, & Pfitzer, 2009). Once activated, Rho can promote the contractile state, and this contractile state lasts for a long time (Norton et al., 2013).

The current study was designed to investigate the pathogenic mechanisms of abnormally contracting sarcomeres in MTrPs, and we analysed changes in cellular morphology, RTK proteins and Rho family proteins in MTrP regions.

## 2 METHODS

### 2.1 Participants

This study was approved by the research ethics committee at Qilu Hospital of Shandong University (KYLL‐2014‐027). All subjects who participated in this study were recruited from January 2018 to January 2019 and provided written informed consent before screening and enrolment. Patients with MTrPs were recruited for the study at the orthopaedics department of Qilu Hospital at Shandong University, Jinan, China. Non‐MPS controls were recruited via advertisements posted on notice boards in the hospital and around the community.

To confirm eligibility, all 73 potential subjects responded to a questionnaire regarding their basic health conditions and all underwent standardized clinical testing, including body mass index calculation and blood pressure measurements. The questionnaire included questions about the intensity of habitual pain in the neck‐shoulder area using a numeric rating scale (0 = no pain and 10 = the most severe pain). For both groups of potential subjects, clinical examinations were performed to evaluate the inclusion and exclusion criteria (see below). All subjects were examined by the same healthcare professional who had more than 20 years of clinical experience in the MPS field to avoid confounding factors caused by different investigators. According to the international consensus on the MTrP diagnostic criteria from 2017 (Gerwin, 2018), the inclusion criteria for the patients recruited into this study were as follows: (a) the presence of a TB; (b) a tender area on the TB; (c) reproduction of the subject's pain by stimulating the TB at MTrPs; and (d) chronic pain lasting at least 1 month. The inclusion criteria for the healthy controls were an age of 18–65 years and the absence of pain. The exclusion criteria for both groups were conditions that might affect pain sensitivity, such as systemic inflammatory diseases, previous neck trauma or surgery in the neck/shoulder area, neuropathic pain, chronic widespread muscle pain conditions, analgesic use within 1 week prior to participation, metabolic disease, high blood pressure, malignancy, pregnancy and a previously failed biopsy.

Finally, seven subjects did not meet the inclusion criteria because the duration of pain experienced was not longer than 1 month, and three subjects met the exclusion criteria related to analgesic use or failed biopsies. Thus, 29 MPS patients and 24 healthy controls were included in this study.

### 2.2 Muscle biopsy

Muscle samples were obtained from the TB of the upper trapezius muscle, including the MTrPs, according to the standard procedure by Bergstrom (Bergstrom, 1975). A disposable SuperCore^™^ Biopsy instrument (Argon Medical Devices, Inc.) was used, which is an improved version of the Bergstrom muscle biopsy system.

First, using the MTrP palpation protocol, the MTrP was identified using the index, middle and ring fingers of the right hand. The painful points on the TB were used to define the location of the MTrP on the superior trapezius muscle. Once the examiner confirmed the position of the MTrP under the pad of the middle finger, a surgical pen was used to mark a point on the surface of the skin to identify the puncture site of the biopsy needle. A 0.5% lidocaine solution in a 1.5 ml volume was used for subcutaneous injection of anaesthetic to minimize pain at the puncture site of the skin. No anaesthetic was injected into the muscle. When the biopsy needle reaches the MTrP, muscle pain can be triggered. The TB was snapped with the thumb and index finger to prevent pneumothorax. The biopsy needle was inserted into the muscle, and, while the local twitch response or pain sensation was triggered, the needle tip was gradually advanced 3 mm (the biopsy instrument had a groove 3 mm from the needle tip), aligning the MTrP with the groove to allow for biopsy collection. In the control group, following subcutaneous infiltration of the anaesthetic, biopsies were taken from the upper trapezius muscle at the midpoint between the 7th cervical vertebra and the acromion in accordance with a previous study (Olausson et al., 2015).

To reduce the sampling error, the biopsy procedures used for all the subjects were performed by an experienced clinician who is a practicing physician with experience in the evaluation and treatment of musculoskeletal disorders and an instructor of these techniques. The biopsied tissues were immediately snap‐frozen in liquid nitrogen‐cooled isopentane and stored at −80°C.

### 2.3 Haematoxylin‐eosin and Masson staining

The muscle specimens were divided into two groups and fixed with formalin. The specimens were subsequently embedded in paraffin wax and sectioned into 3 mm sections, which were mounted on slides and stained with haematoxylin‐eosin (H&E) and Masson's trichrome stains. The sections were dehydrated in a graded ethanol series (70%–100%) and xylene, then cover‐slipped.

Images were captured with a BX53 microscope (Olympus Corporation) using a Q Imaging Micro Publisher camera (Abingdon) and Q Capture software (VayTek Inc.).

### 2.4 Differential expression analysis and target gene prediction

RNA isolation, library preparation and sequencing analysis were performed as previously described. Library construction and sequencing were performed at Shanghai Sinomics Corporation. Differences in gene or digital transcript expression were determined using Cuffdiff statistical analyses (Fatica & Bozzoni, 2014). Genes or transcripts with differing expression levels between groups at *p* < 0.05 were considered to be differentially expressed. Target gene prediction of long non‐coding RNAs (lncRNAs) was performed as previously described (Trapnell et al., 2010). The coding genes 10 kb upstream and downstream of the lncRNAs were searched as the target genes in the *cis* orientation.

### 2.5 lncRNA/mRNA co‐expression network

To study the relationship between lncRNA and mRNA, we calculated the co‐expression relationship between lncRNA and mRNA according to dynamic changes in gene expression signal values, and we determined the expression regulation relationship and direction between genes needed to construct the gene expression regulation network. By using this co‐expression network, we were able to analyse genetic regulatory ability and identify the core regulatory genes that differed between the study groups. The co‐expression network was constructed using Cytoscape open source software.

### 2.6 RTK phosphorylation analysis

Antibody arrays of phosphorylated RTK (p‐RTK) were performed using the RayBio Human Phospho Array Kit (catalogue no. AAH‐PRTK‐G1) which was purchased from Ray Biotech, Inc. The assay for the p‐RTK array was conducted according to the manufacturer's instructions. In brief, 100 µl of 1× blocking buffer was added to each well and incubated at room temperature with gentle shaking for 30 min. All of the blocking buffer was decanted from each well, then 100 µl of each sample was added to the appropriate wells. The arrays were incubated with the samples at 4°C overnight. The samples were decanted from each well and washed three times with 100 µl of 1X wash buffer I at room temperature with gentle shaking. Then, 100 µl of 1× biotin‐conjugated anti‐phosphotyrosine antibody was added to each well. The arrays were incubated at room temperature with gentle shaking for 2 hr. After the subarrays were washed three times, 100 µl of 1× fluorescent dye‐conjugated streptavidin was added to each one. The arrays were then washed three times and completely dried. The signals were imaged using a laser scanner.

### 2.7 Immunohistochemistry

Immunohistochemistry was used for streptavidin‐biotin labelling. Paraffin‐embedded sections were heated at 68°C for 2 hr, separated in xylene, and rehydrated in graded ethanol at room temperature. The sections were microwaved for 15 min in EDTA buffer (pH 8). After being rinsed twice in phosphate‐buffered saline (PBS), the slices were placed in a wet chamber and incubated in 3% hydrogen peroxide for 10 min. After being washed with PBS three times, the sections were incubated with 10% normal goat serum at 37°C for 30 min, then diluted with the following antibodies at 4°C overnight: anti‐Eph receptor B1 + Eph receptor B2 (phospho Y594 + Y604) antibody (Abcam ab61791 1:300), anti‐RhoA (Abcam EP487Y 1:200), anti‐Rac1 (Abcam ab64533 1:150) and anti‐Cdc42 (cell division control protein 42; Abcam ab180785 1:300). After being washed with PBS three times, the slices were incubated with biotin‐labelled goat anti‐rabbit serum at 37°C for 30 min, then treated with a horseradish peroxidase‐labelled streptavidin complex at 37°C for 30 min (Origene PV9000). The reaction products were observed with a diaminobenzidine hydrochloride substrate kit. Finally, after weak haematoxylin staining, the sections were dehydrated and covered. The negative control group was incubated with PBS instead of a primary antibody. Images were obtained with a BX53 microscope (Olympus) and a Q‐Imaging Micropublisher camera (Abindon).

### 2.8 Western blot analysis

Protein expression in muscle was evaluated by Western blot analysis as previously described (Zhang, Jin, Zhu, & Qi, 2020). In brief, p‐EphB protein expression was assessed using a specific antibody (Abcam ab61791 anti‐Eph receptor B1 + Eph receptor B2 (phospho Y594 + Y604); 1:1,000). The total proteins from the muscle tissues were separated by 10% SDS‐PAGE and transferred to a PVDF membrane, which was blocked with 5% non‐fat milk and incubated with primary antibodies at 4°C overnight. The transferred blots were developed with a chemiluminescent reagent (Millipore).

### 2.9 Statistical analyses

The data were analysed using GraphPad Prism 7.0 statistical software. The quantitative data are presented as the means ± *SD*. The Shapiro–Wilk test revealed that the data were non‐normally distributed; therefore, the data were analysed using non‐parametric tests. Differences among three groups were tested using one‐way ANOVAs; when a significant difference was detected, the least significant difference method was used for comparison between groups. *p* < 0.05 was considered statistically significant.

The number of subjects required for this study was based on previously reported levels of interstitial lactate in the trapezius muscle of healthy controls and patients with chronic trapezius myalgia (Rosendal et al., 2004). The type I error *α* of the hypothesis test was 0.05 (two‐tailed), the type II error *β* was 0.1, and the sample size ratio of the two groups was 1:l. Using Power Analysis and Sample Size version 11.0, the sample size was calculated according to the sample size formula:

$$n_{1}=n_{2}=2{\left[\frac{(t_{\alpha }+t_{\beta })s}{\delta }\right]}^{2}.$$

The minimum required sample size calculated for each group was three subjects. Twenty‐nine MTrP patients and 24 healthy controls were included in this study. For more information on the number of subjects included in each experiment, please see Supporting Information.

## 3 RESULTS

### 3.1 Muscle histology in human MTrPs

**FIGURE 1:**
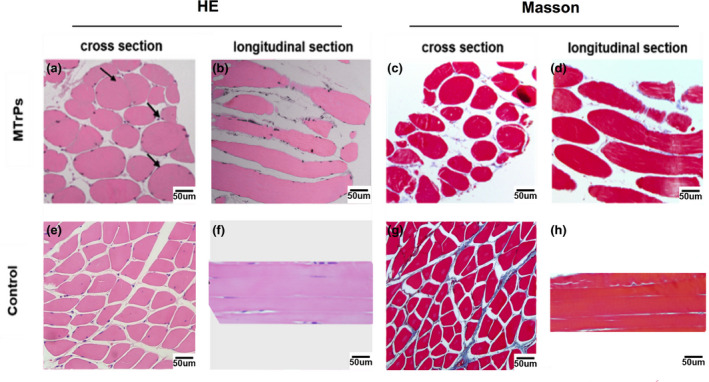
Histology of human trapezius muscle in myofascial trigger points (MTrPs) samples and control samples. Muscle was stained with haematoxylin‐eosin (H&E) (a,b,e,f) and Masson stain (c,d,g,h) in MTrP samples (a–d) and control samples (e–h). Normal muscle cells were observed in the control group (e). Enlarged, round, contracted muscle (black arrow) was observed in MTrPs patients (a)

Following H&E staining, the muscle fibres were observed under a microscope. In the control group, the fibres were uniform in size, polygonal in shape and regular in arrangement in cross‐sectional and longitudinal spaces (Figure 1e,f). However, the muscle fibres in the MTrPs displayed annular or elliptical muscle fibres of different sizes in cross‐sectional spaces (Figure 1a). Continuous expansion of the pyramidal muscle fibres was observed in the longitudinal sections. The muscle fibre space of the transverse section and longitudinal section increased (Figure 1b). Under Masson staining, in the control group, the muscle fibres were uniformly stained red. They were arranged homogeneously with no blue staining, and only a small amount of blue stain was found in the stroma (Figure 1g,h). In the muscle fibres of the MTrPs (Figure 1c,d), the sarcomere arrangement was uneven, the muscle fibres were stained red, no blue staining was observed in the cytoplasm, and only a small amount of blue stain was found in the stroma.

### 3.2 RTK and EphB (1, 2, 3) phosphorylation are up‐regulated in human MTrPs

**FIGURE 2:**
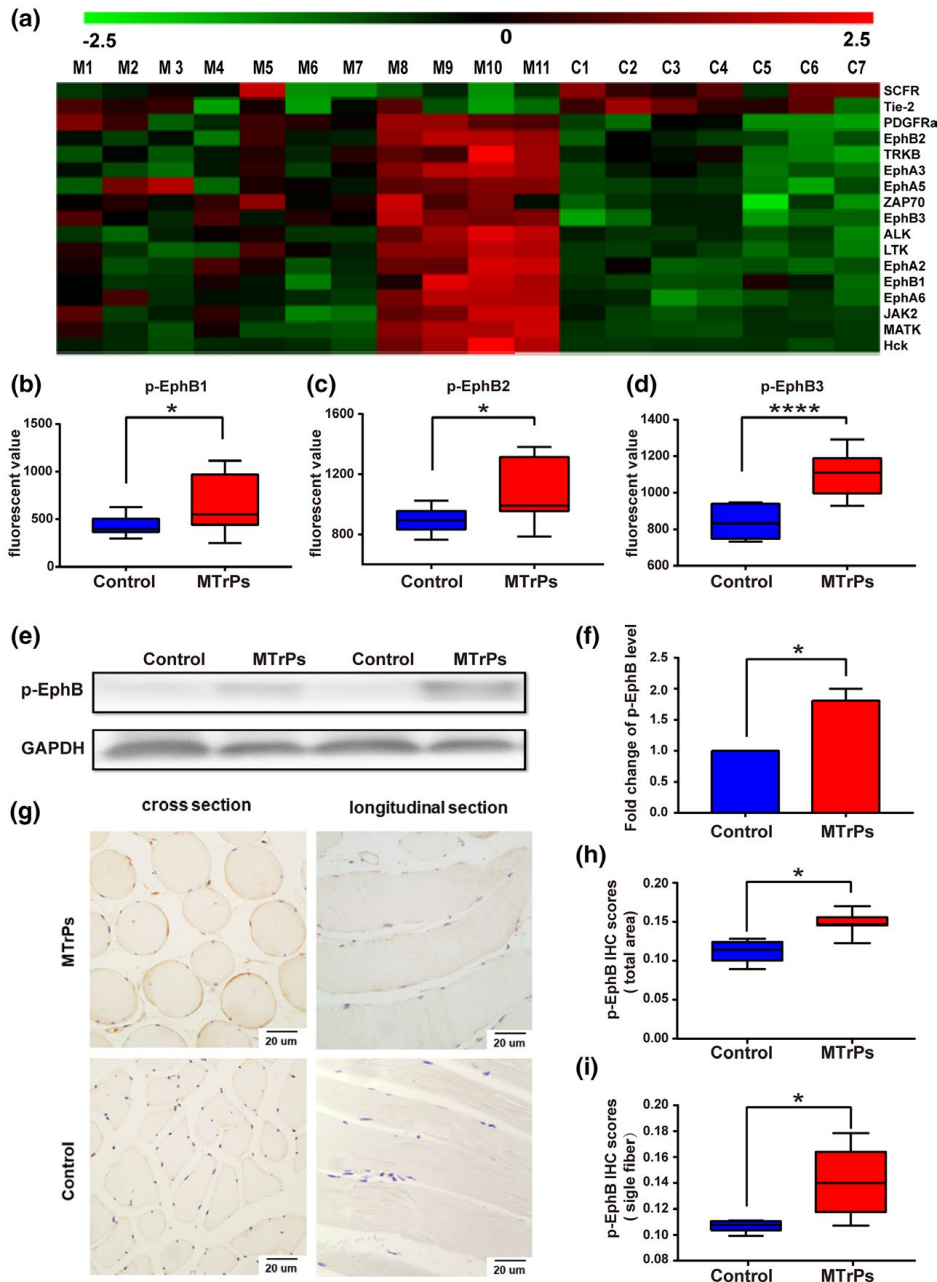
Receptor tyrosine kinase (RTK) phosphorylation expression in MTrPs. (a) RTK proteins with different phosphorylation levels between MTrP (*n* = 11) tissues and control group tissues (*n* = 7). (b–d) Levels of p‐Eph in MTrP and control group samples. The levels of p‐EphB1, p‐EphB2, and p‐EphB3 were significantly higher in MTrP patients than in controls. (e) Representative bands of p‐EphB expression in control and MTrPs muscle tissues. (f) Quantification of the average means optical density for p‐EphB in the control group and MTrP group. (g) Representative images of p‐EphB expression in control muscle tissues and MTrPs (×400). (h,i) IHC score of the total area (h) and single fiber (i) for p‐EphB in the control group and MTrP group. IHC staining score was formulated as integrated optical density/size. Data are presented as *M* ± *SEM* or as medians and interquartile ranges (25th and 75th percentiles). IHC, immunohistochemistry; MTrPs, myofascial trigger points. *p < 0.05, ****p < 0.0001

The phosphorylated protein expression array of MTrP and control tissues was analysed. Among the 71 tyrosine kinases, 15 proteins, including TrkB and EphB, had increased phosphorylation levels in MTrP tissue relative to the levels in control tissue, and two proteins, mast/stem cell growth factor receptor and Tie2, showed decreased levels in MTrP tissue (Figure 2a). The expression of p‐EphB1 (*p* < 0.05), p‐EphB2 (*p* < 0.05) and p‐EphB3 (*p* < 0.001) was higher in MTrPs than in normal tissue (Figure 2b–d).Western blot analysis confirmed that relative to the expression in the control group, the expression of the p‐EphB protein in MTrPs was significantly up‐regulated (Figure 2e,f; **p < 0.01). Immunohistochemical analysis showed an increase in p‐EphB levels on the muscle cell membrane of contractile sarcomeres in MTrPs (Figure 2g–i). These results suggest that the expression of p‐EphB is up‐regulated in the muscle tissue of MTrPs.

### 3.3 lncRNA‐mRNA co‐expression network

**FIGURE 3:**
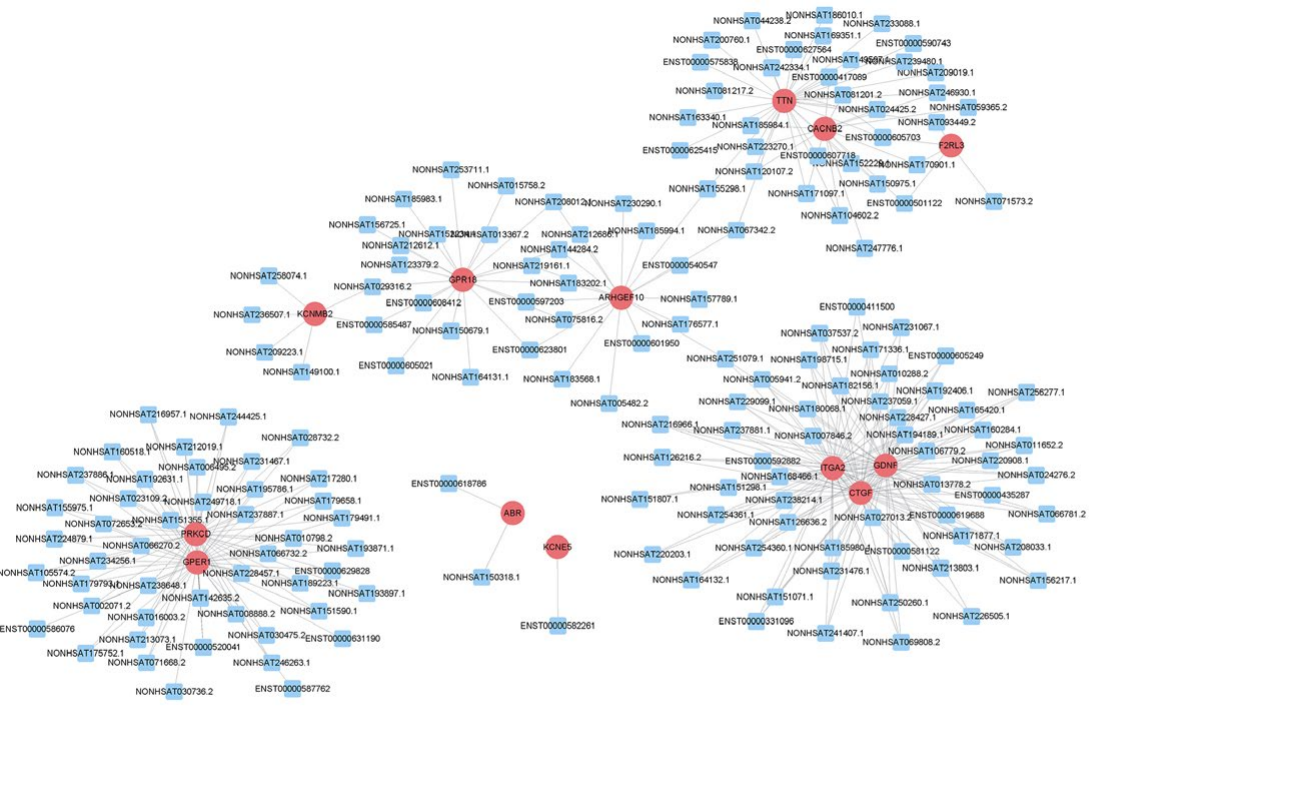
Long non‐coding RNA (lncRNA)‐mRNA network analysis of the muscle contraction signaling transduction (Pearson's coefficient > 0.99). Red ovals represent mRNAs, blue rectangles represent lncRNAs, a line represents the correlation

To predict gene functions, we constructed an lncRNA‐mRNA co‐expression network and performed a pathway analysis (Figure 3). A total of 176 lncRNAs and 13 mRNAs (Pearson's coefficient > 0.99) were identified during muscle contraction signal transduction. The co‐expression network consisted of 115 network nodes and 350 connections.

### 3.4 RhoA and Rac1, but not Cdc42, are activated in the contractile sarcomeres of MTrPs

**FIGURE 4:**
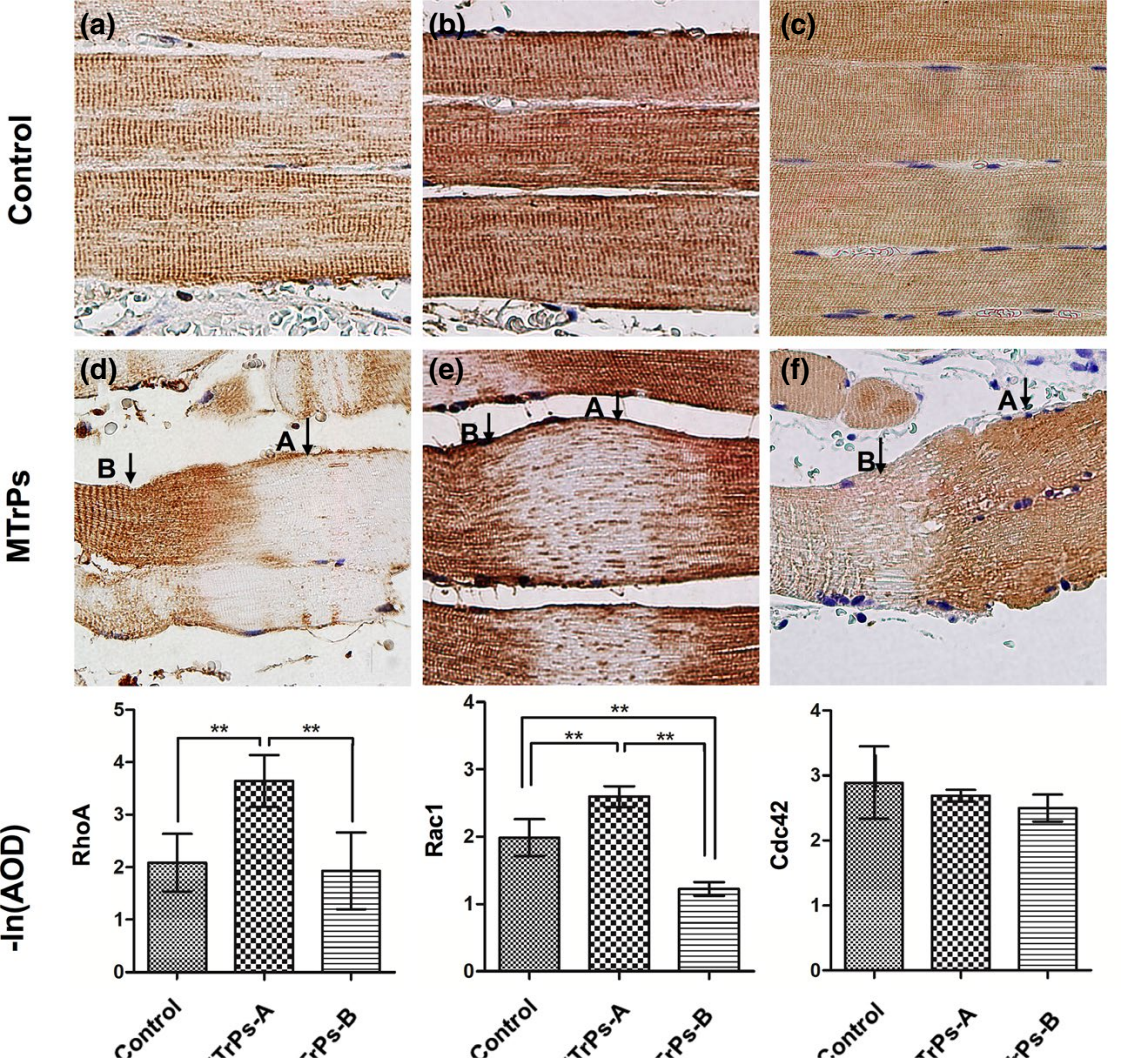
RhoA and Rac1, not Cdc42, transfer membrane activation in the contractile sarcomeres of MTrPs. (a–c) In the control group, Rho A, Rac1, and Cdc42 were expressed evenly in the muscle sarcomere. (d) In the MTrP group, in site A, the expression of RhoA in cytoplasm was decreased, with RhoA having migrated to the cell membrane, and the expression in site B was increased. (e) In the MTrP group, in site A, the expression of Rac1 in cytoplasm was decreased, with Rac1 having migrated to the cell membrane, and the expression in site B was decreased. (f) The expression of Cdc42 in sarcomeres of the MTrP group was uneven but have no consistency. (A) The abnormal contractile site; (B) the elongated site adjacent to site A. **p < 0.01

In the control group, RhoA, Rac1 and Cdc42 were uniformly expressed in the muscle fibres located in the cytoplasm of the muscle (Figure 4a–c). In MTrPs, the expression of RhoA (Figure 4d) and Rac1 (Figure 4e) in the cytoplasm of the contractile site cells (site A) was significantly decreased (*p* < 0.01 vs. control group); labelling was observed in the cell membrane and perimembrane cytoplasm. In the elongated portion, the expression of RhoA and Rac1 in the cytoplasm of cells in a relaxed position (site B) was increased relative to that of site A(*p* < 0.05), but no difference was found between the control group and the MTrP group (*p* > 0.05).The expression of Cdc42 was uneven and irregular in the sarcomeres (Figure 4f).

## 4 DISCUSSION

This study revealed the following: (a) The abnormally contracting sarcomeres in the MTrP regions showed enlarged, round fibres without inflammatory cells and fibrosis; (b) The lncRNA‐mRNA network analysis using Smart‐seq demonstrated that muscle contraction signalling was activated in the MTrP regions; (c) In the MTrP group, relative to the control group, 15 proteins in the RTK family were found to be significantly up‐regulated, and two proteins were found to be significantly down‐regulated, with a particular increase in EphB1/EphB2 expression on the muscle cell membranes of the abnormally contracting sarcomeres; and (d) RhoA and Rac1, but not Cdc42, were activated at the abnormal sarcomeres with different degrees of transmembrane activity.

Few studies have revealed the histological changes that occur in MTrP regions, especially in MPS patients. In our research, the muscle fibres in the MTrP site exist in a pathological contractile state and gradually form contracture nodules. Simons et al. (1999) have compiled anatomical charts of MTrPs, describing the muscle fibres of MTrPs as small and large round fibres in cross‐sections, and they hypothesized that large fibres were the middle parts of the contractile sarcomeres, whereas the small fibres were the distal parts. The appearance of the muscle fibres in MTrPs in our research supports this deduction.

Some studies have revealed the biochemical environment of active MTrP sites, including concentrations of cytokines, neuropeptides and catecholamines, which differ from those of normal muscle areas or latent MTrP sites (Shah et al., 2008). Large gaps exist between contracture nodes, which may contain a large number of biological mediators. In several animal models, such as the eccentric‐based exercise model described by Huang, Ye, Zhao, Lv, and Tang (2013) and a model developed by Hayashi et al. (2011) with eccentric contraction, extensive inflammatory cell infiltration has been observed in areas diagnosed as MTrPs; however, in the present study, in the MTrP tissue sections of MPS patients, obvious inflammatory cell infiltration was not observed. The increase in biologically active substances might be due to the fact that skeletal muscle is a secretory tissue, releasing cytokines and other peptides with autocrine, paracrine and endocrine effects (Pedersen & Febbraio, 2008, 2012). A previous study showed that skeletal muscle can secrete various biochemicals during muscle tissue injury and repair (Pedersen, 2011).

The muscle pain in MPS patients might be derived from fibrotic nodules (Fischer, 1988). We used Masson trichrome staining to detect fibrosis at the MTrPs, which revealed the distribution of collagen and muscle fibres in the tissue. This staining method is frequently used to reveal the degree of fibrosis. In the current research, Masson staining revealed no significant fibrosis in MTrP tissues relative to control tissues, which is inconsistent with previous studies suggesting that MTrPs in TBs or nodules are caused by fibrotic scar tissue (Fischer, 1988).

Considerable evidence supports the view that activation of the RTK family and related downstream pathways are conducive to the development of peripheral sensitization (Cao et al., 2008; Rivat et al., 2018). In the present study, detection of RTK phosphorylation changes in MTrPs using antibody microarrays revealed that among the 71 RTK family members, 15 proteins displayed increased levels of phosphorylation, while two proteins showed decreased levels in the MTrP group. These results show that the development of MTrPs and the up‐regulation of RTK proteins are highly correlated. As mentioned above, the primary characteristic of MTrPs is abnormally contractile sarcomeres. The RTK proteins are important for actin cytoskeletal remodelling and muscle contraction (Huang et al., 1997; Norton et al., 2013). We focused on the Eph family, the largest member of the RTK family (Dorsher, 2008), which plays critical roles in regulating cell shape, adhesion, migration and positioning during developmental processes (Lai & Ip, 2003). In our research, the expression of EphB1/EphB2 was significantly increased in the membranes of enlarged contractile sarcomeres. Eph receptors are expressed in many organs, including muscle tissue. Eph can facilitate cross‐linking by actin filaments and can regulate remodelling of the actin cytoskeleton (Shamah et al., 2001; Tolias et al., 2007), and actin is vital for the stability of Ach receptor (AChR) clusters in muscle cells (Dai, Luo, Xie, & Peng, 2000). A recent integrated hypothesis (Gerwin et al., 2004) on MTrPs indicates that dysfunctional neuromuscular end‐plates releasing Ach might be responsible for the TB phenomenon. Botulinum toxin treatment is based on this principle (Ernberg et al., 2011). However, previous studies have reported that injection of botulinum toxin A into the MTrP area had no effect on mechanical pain thresholds or pain intensity (Ernberg et al., 2011; Richards, 2007). Furthermore, reports indicate that intramuscular injection of a small amount of acetylcholinesterase inhibitor resulted in muscle fibre breakage and contractile nodules at the injection site (Mense et al., 2003) that were different from the abnormally contractile sarcomeres in MTrPs biopsied from MPS patients. This abnormal contraction of the sarcomeres of MTrPs may be related to abnormal protein activity in the local muscle.

A previous study demonstrated (Carmona, Murai, Wang, Roberts, & Pasquale, 2009) that Eph receptors participate in the maintenance of postsynaptic morphology. Activation of EphB2 arrests tau hyperphosphorylation. Hyperphosphorylated tau cannot promote microtubule polymerization. Thus, activation of EphB2 will enhance microtubule formation, which is consistent with the ideas presented by Jafri (2014). EphB2 can also activate spine morphogenesis and Eph signalling, inducing activation of RhoA and leading to local actin rearrangement and retraction of the spines (Penzes et al., 2003). Phosphoinositide 3‐kinase (PI3K) is regulated by direct interactions with RTKs and their tyrosine phosphorylated substrates, and it binds activated Rho family members, which can produce a persistent contraction (Bresnick & Backer, 2019).

**FIGURE 5:**
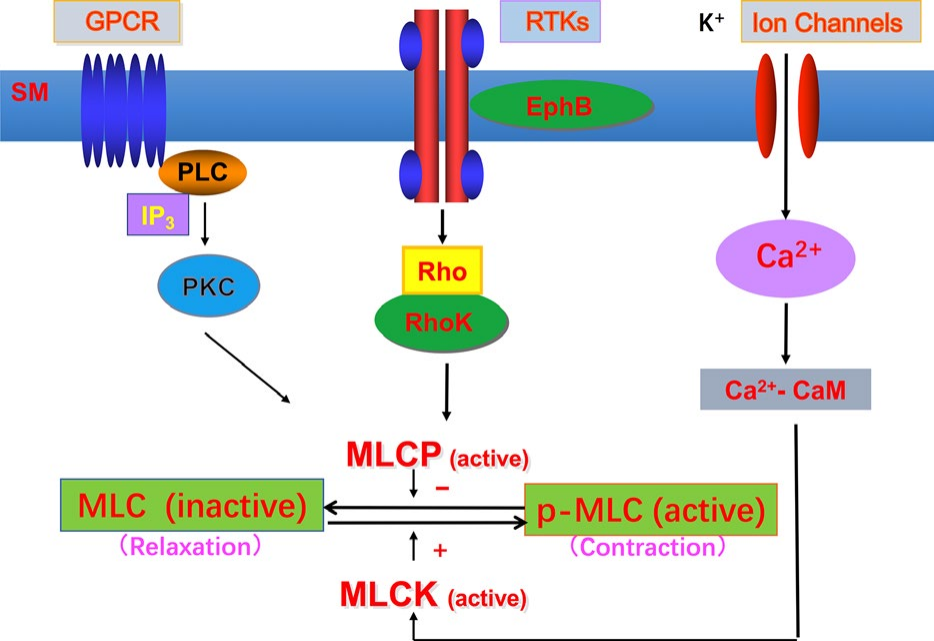
Schematic diagram of skeletal muscle contraction pathway mediated by RTKs. MLC, myosin light chain; MLCK, myosin light chain kinase; MLCP, myosin light chain phosphatase; p‐MLC, myosin light chain phosphorylation; RTKs, receptor tyrosine kinase; GPCR, G protein‐coupled receptor; PLC, phospholipase C; PKC, protein kinase C; SM, skeletal muscle

To further explore the possible mechanisms associated with abnormally contractile sarcomeres and to obtain more information about the signalling pathways related to differences in muscle contraction between patients with MTrPs and healthy controls, we compared differences in lncRNAs and mRNAs in muscle tissue. Understanding differences in these lncRNA‐mRNA co‐expression networks will contribute to a better understanding of the pathogenesis of MTrPs, though further research is needed. The co‐expression of lncRNA and mRNA indicated that some key regulatory proteins of Rho kinase, such as GTPase‐activating protein (ABR) and Rho guanine nucleotide exchange factors (RhoGEFs), and other lncRNA or mRNA associated with muscle contraction signalling were up‐regulated, with only a few being down‐regulated. RhoGEFs can increase the exchange rate of GDP/GTP (Puetz et al., 2009), increase the proportion of Rho‐GTP, and activate Rho kinase and transmit the signal downstream. To date, research on the Rho family has primarily focused on the contraction of smooth muscle, and no reports have explored the Rho family's role in regulating skeletal muscle contraction. In the present research, members of the Rho (RhoA and Rac1, but not Cdc42) family were activated in the abnormal contraction site of MTrPs, with different degrees of transmembrane activity. The mechanism of Ca^2+^ sensitivity is regulated by RhoA/Rho kinase to prevent myosin phosphatase light chain dephosphorylation from maintaining force generation (Shibata et al., 2015), which can cause the muscles to contract continuously for a long time. A recent study showed that this signal is also expressed in skeletal muscle (Ford‐Speelman, Roche, Bowman, & Bloch, 2009; Zhang, Huang, & Gunst, 2012). Eph participates in cell–cell interaction‐mediated RhoA activation, which regulates the contractility of vascular smooth muscle (Wu et al., 2012). These findings support the hypothesis that the Rho family may play a key role in the muscle contractility of MTrPs (Figure 5).

MTrPs are associated with muscle areas containing stiff nodules detectable by palpation. This stiffness is believed to be caused by hypercontracture of local sarcomeres (Simons & Stolov, 1976). Our histological examination of muscle biopsies from MTrPs revealed structural evidence of contracted sarcomeres in human skeletal muscle, which is consistent with hypotheses related to trigger point formation. Sustained contractile activity leads to an increase in metabolic stress and reduced blood flow, which are likely responsible for secondary changes that lead to the persistence of MTrPs. In addition, continuous contractile activity, metabolic changes, and cellular stress can trigger increased release of myokines, inflammatory cytokines and neurotransmitters, which undoubtedly contribute to these MTrPs and MPS. We found abnormal activation of EphB1/EphB2 and RhoA/Rac1 in contractile muscle fibres; changes in these pathways may facilitate actin filament cross‐linking and regulate remodelling of the actin cytoskeleton (Shamah et al., 2001; Toliaset al., 2007). These actions lead to continuous contraction of muscles and the formation of TBs where MTrPs are localized. In addition, considering the role of Eph and/or Rho in peripheral sensitization (Cao et al., 2008), activation of Eph and/or Rho may be involved in the mechanistic formation of MTrPs, so these signalling molecules might be promising therapeutic targets for MPS.

### 4.1 Limitations

A number of limitations exist in this study. Firstly, we were unable to confirm that the biopsies were collected from a precise area, even when we tried to biopsy the MTrP site. Secondly, given the difficulty of collecting human samples, the sample size was small and the statistical power was low, leading to an increased probability of type II error. Furthermore, the weight of the needle biopsy tissue was approximately 3–8 g, which is not sufficient for future research to explore and verify protein changes. In addition, a credible animal model is needed to verify the functions of EphB1/EphB2 and RhoA/Rac1.

## 5 CONCLUSION

In conclusion, our research demonstrated that EphB1/EphB2 and RhoA/Rac1 might mediate or participate in the abnormal contraction of sarcomeres in MTrPs, which showed no inflammatory cell infiltration or fibrosis. This research provides a promising therapeutic target for MPS. Next, functional verification of related proteins and pathways should be carried out.

## CONFLICT OF INTEREST

None declared.

## AUTHORS’ CONTRIBUTIONS

Feihong Jin recruited all the subjects and designed, performed, and analysed the histology experiments to which Yaqiu Guo, Zi Wang, and Xin Pan contributed. Feihong Jin designed and performed the antibody arrays and RNA sequencing to which Ahmed Badughaish contributed. Feng Qi and Li Zhang conceived the project and supervised all the experiments.

## Supporting information

Supplementary Material

## Data Availability

The authors declare that all data supporting the findings of this study are available within the paper and its Supporting Information files or from the authors upon request.
