# Supplementary figures and images for: The pathophysiological nature of sarcomeres in trigger points in patients with myofascial pain syndrome: A preliminary study
Source: Eur J Pain. 2020 Sep 10;24(10):1968–78. doi: 10.1002/ejp.1647 (PMC7693045; doi:10.1002/ejp.1647)

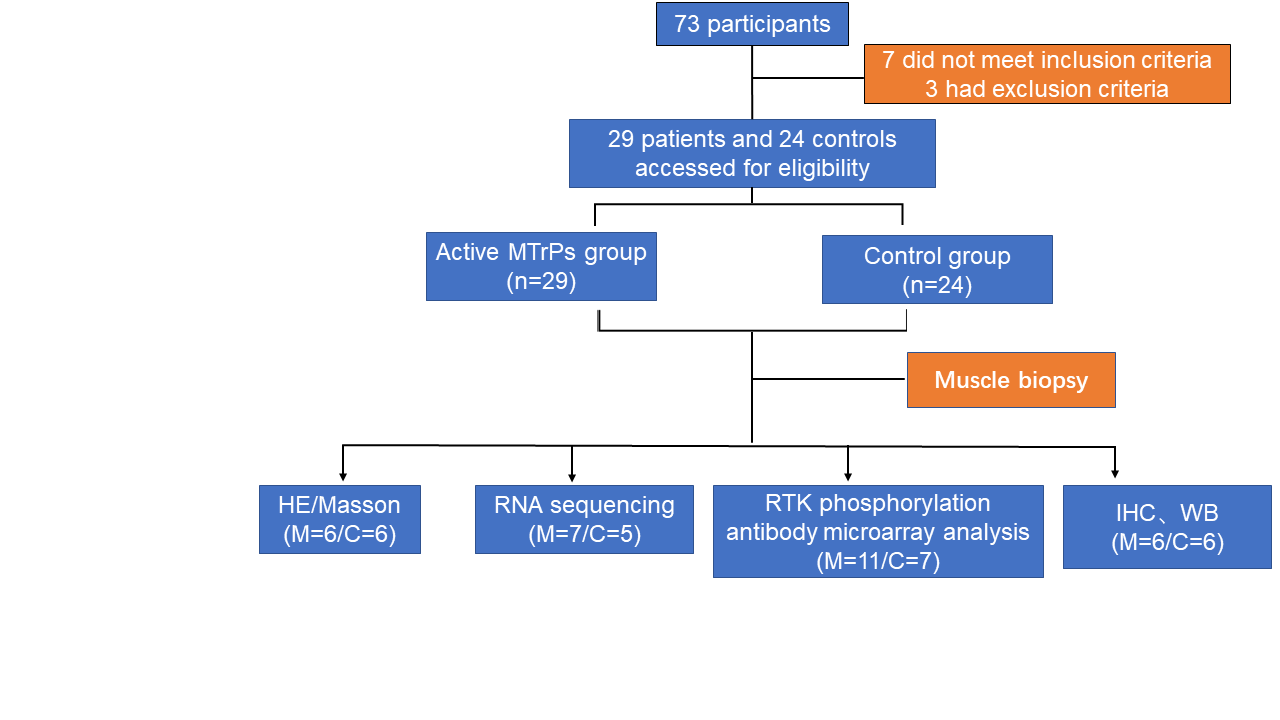

Supplement: Supplementary file 1 — Supplementary Material [file EJP-24-1968-s001.docx]
